# A pricing model for group buying based on network effects

**DOI:** 10.1371/journal.pone.0211109

**Published:** 2019-01-24

**Authors:** Guanqun Ni

**Affiliations:** College of Management, Fujian Agriculture and Forestry University, Fuzhou, Fujian, China; Northeastern University, CHINA

## Abstract

Group buying (GB) is a popular business model in e-commerce. With the rise of online social media, the positive network effect of buying with others is more important than price discount for consumers to choose GB. However, the negative network effect of GB is also significant for some consumers. In this paper, we classify consumers into two segments considering both positive and negative network effects, and three possible sales strategies as well as their optimal decisions on price are presented. We find that GB strategy dominates individual buying (IB) strategy when the positive network effect is sufficiently high or the proportion of consumers with low valuation is relatively large. We also find that MIX strategy offering both IB and GB is always better than IB, while the relationship between MIX and GB is depending on actual market situations. Some other managerial insights are also discussed.

## 1 Introduction

Group buying (GB) is a form of selling under which consumers are encouraged to buy together and discounts are offered based on consumers’ aggregated purchasing quantity [1]. This practice is observed in a variety of product categories, ranging from consumer electronics and furniture to dental services and museum visits [2]. For consumers, the most compelling reason to participate in GB is financial, specifically getting low price due to discounts negotiated between GB consumers and sellers. Low price, however, is not GB’s only goal. Extant research has shown that shopping with someone enhances the overall shopping experience and that the presence of other persons in a GB situation is likely to have an influence on the decision to make a purchase [3–5]. Also, pointed out by Wen et al. [6, 7], the decision-makers’ risk preference and thus decisions on purchase are more likely to be influenced under uncertain environment. One of the most distinctive features of online GB is that online shoppers cannot directly observe and experience a product and can only acquire product information from the pictures and text posted on the retailer’s website. Thus, a gap sometimes occurs between the actual product and consumers’ expectation, which in turn leads to the uncertainty risk of product quality [8, 9]. Under such a situation, the presence of other buyers in shopping instances can reduce a consumer’s uncertainty risk of product quality and thus enhance a consumer’s perceived quality of product [10]. In the field of GB, this kind of impact of other buyers’ presence on one’s perceived quality of product is called the positive “network effect” in the literature [11]. A positive network effect also arises for consumers in a GB option when buying with friends and family because of information exchange, affirmation of choice, and lower cognitive load when deciding what to buy [5]. With the rise of online social media and social networks, the positive network effect is even more important than price discount.

Meanwhile, there is some inconvenience inherent in buying with others, such as delays in manufacturing or transaction, limitations in purchasing quantity, and risk issues [12]. Luo et al. [13] also point out that, compared to individual buying (IB), GB requires some extra effort on the part of consumers. In practice, the GB sellers usually set a minimum purchase threshold to activate a sale, which is also known as the packet size. When demand is lower than the packet size, consumers cannot activate the channel of purchasing via GB. As a consequence, the waiting cost will possibly increase with the packet size. From the perspective of shopping experience, Borges et al. [14] argue that when the consumer identifies highly with a shopping environment, s/he finds more enjoyment and value shopping alone than with other buyers. Kumar and Rajan [15] also argue that social coupons are not always bringing positive network effects to consumers, particularly when consumers are seeking a one-time deal. Generally, it is sometimes inconvenient and/or unenjoyable when buying with others in GB, and thus we call this kind of impact of other buyers’ presence “negative network effect”. The negative network effect is also called “negative externality” in the literature [11].

Some recent studies investigate the network effects of other buyers’ presence in GB. Liang et al. [16] study the effects of strategic customer behavior on GB from a price-discrimination perspective. They argue that information update has a positive effect on customer surplus and the GB success rate. Focusing on the negative network effects, Ni et al. [17] divide consumers into two segments according to their communication costs in GB and find that offering a GB option might decrease seller’s profit in several circumstances. Based on a game theoretic formulation, Ni et al. [18] argue that GB is more efficient when the communication cost is smaller and there is also an upper bound of communication cost for the seller to offer a GB option.

Considering both positive and negative network effects, Zhang et al. [11] argue that when the positive network effects from participation are higher relative to the cost externality, a firm is more likely to favor a GB strategy over an IB strategy. In some cases, the negative network effects in buying within a group may surpass the positive network effect of buying with a social circle. Under such circumstances, the seller may be forced to sell only to individuals. Similarly, in this paper, we also find that although consumer utility can be enhanced by the positive network effect in GB, offering a GB option can lead to lower revenues in some cases. Differently from their work, we assume that consumers are heterogeneous and the market is composed of two segments of consumers; hence, we obtain more general results for some cases.

A number of extant researches show that GB dominates IB when consumers are sufficiently heterogeneous [2, 19–21]. More recently, Tran and Desiraju [22] classify consumers into two segments by their valuations, wherein the valuations of consumers in the *H* segment are systematically higher than those of consumers in the *L* segment. They argue that GB dominates IB when the *H* segment is relatively large or when the price sensitivity of consumer in the *L* segment is relatively high. However, Tran and Desiraju [22] only consider the negative network effect without the positive network effect of GB and assume that the negative network effect is more significant for the *H* segment than for the *L* segment. Differently from their work, we consider the negative network effect as well as the positive network effect of GB and further assume that the positive network effect is less significant for the *H* segment than for the *L* segment, and thus we generalize their model to some degree.

The current study focuses on GB and IB options of a seller and identifies the optimal selling strategies for a monopolist. We investigate whether the seller find it optimal to sell product via GB or IB and how offering a GB option influences the pricing and profits of the seller. Particularly, this study addresses the following questions: (1) What is the impact of positive and negative network effects through the GB mechanism? (2) What is the optimal selling strategy for the seller when deciding to offer either the GB option or IB option? (3) What are the conditions under which a pure GB, a pure IB, or a mixed strategy is the most profitable?

Our goal in the current study is to advance the understanding and application of GB by explicitly accounting for the utility from shopping with others. In line with this goal, we develop a mathematical model to capture the consumer’s utility from shopping with others and the seller’s benefit from selling via different options. Our result shows that both the network effect and consumer heterogeneity play a significant role in choosing optimal strategy. In particular, offering only GB dominates offering only IB when the positive network effect is sufficiently high or the proportion of low valuation segment is relatively large. We also find that offering both options simultaneously can improve the seller’s revenue compared to offering only IB option.

The rest of this paper is organized as follows. In Section 2, we introduce our model for the market. We compare three pricing strategies (i.e. IB, GB, and MIX) and derive the optimal pricing strategies for the seller in Section 3. Section 4 presents some numerical examples to verify the insights derived in this research. Finally, the concluding remarks are given in Section 5.

## 2 The model

Consider a market served by a monopoly seller who sells a unique product. To focus on the influence of network effects, we assume that the market is in complete information symmetry and consists of two consumer segments, denoted by *H* and *L* respectively. Proportion *m* of consumers belong to *H* segment and they derive a valuation *v*_*H*_ for the product, which is uniformly distributed between *c* and *b* + *c*. Proportion 1 − *m* of consumers belong to *L* segment whose valuations are systematically lower than those of consumers in *H* segment, and thus we assume that consumers in *L* segment derive a valuation *v*_*L*_, which is uniformly distributed between 0 and *b*, for the product. Here, *c* captures the difference of valuations between the two populations. Without loss of generality, we assume that ach consumer is interested in buying a single unit of product. In order to avoid trivial results, we additionally assume 0 < *c* < *b* throughout this paper.

For the seller, there are three possible pricing strategies to choose from: (i) sell only to individuals (IB); (ii) sell only to group (GB); or (iii) offer both mechanisms (MIX). Let *p*_*I*_ be the unit price and *q*_*I*_ be the quantity sold through IB. Let *q*_*G*_ be the threshold quantity adopting GB at unit price *p*_*G*_. The seller’s objective is to maximize revenue with respect to the possible decisions on *p*_*I*_, *p*_*G*_, and *q*_*G*_.

Under the IB mechanism, following [22], we define the net utility of consumer by the difference between valuation and price, and assume that only those whose net utility exceeds 0 will make the purchase. Therefore, consumers in *H* and *L* segments pay the price *p*_*I*_ through IB and obtain the net utilities equal to *v*_*H*_ − *p*_*I*_ and *v*_*L*_ − *p*_*I*_, respectively.

Under the GB mechanism, there are two features separating GB from IB. First, the positive network effects are idiosyncratic and are conditional on the number of participants. Moreover, due to buying with friends, a positive network externality from shopping with others will be added to the utility of the consumer [5, 10]. This value is heterogeneous across consumers and is formally expressed as *β*_*i*_*q*_*G*_ for *i* = *H*, *L* where *β*_*i*_ captures the importance of buying with others through GB. The positive network effect is captured by the fact that this additional utility increases with the size of consumers who buy under the GB option (*q*_*G*_). As laid out in Section 1, there are a number of reasons for the existence of positive network effects in consumer shopping environments, including collective support and information exchange (e.g. [4, 5, 14]).

Second, the GB option is less convenient than the IB option. As stated in Section 1, the GB sellers usually set a minimum group size to activate a sale, and thus consumers have to wait until the minimum group size is reached. Given a larger required group size, the interested consumers have to wait more time. Otherwise, there might be no sufficient number of consumers to activate the GB option. That is, the waiting cost will possibly increase with the required group size. Besides, when the consumer identifies highly with a shopping environment or is seeking a one-time deal, as confirmed in the literature (e.g. [14, 15]), s/he finds more enjoyment and value shopping alone than with other buyers. Generally, it is inconvenient and/or unenjoyable when buying with others in GB, and thus we call this kind of impact of other buyers’ presence “negative network effect”. As explained by Zhang et al. [11], the negative network effect is captured by the fact that this additional (negative) utility increases with the size of consumers who buy under the GB option (*q*_*G*_), and thus we express the negative network effect as *α*_*i*_*q*_*G*_ for *i* = *H*, *L* where *α*_*i*_ captures the inconvenience of buying with others through GB. Negative network effects exist for consumers who use GB, and an individual is exposed to higher levels of negative effect in a transaction as the buying group gets larger.

In practice, consumers in *H* segment usually identify the product as well as the shopping environment more highly than those in *L* segment. Thus, following Borges et al. [14], we argue that *α*_*H*_ ≥ *β*_*H*_ and *α*_*L*_ ≤ *β*_*L*_, which means that consumers in *H* segment are more sensitive to the negative network effect while consumers in *L* segment are more sensitive to the positive network effect.

Compared with IB, the net utility of consumers in GB should further add the positive network effect and subtract the negative network effect. Let *α* ≔ *α*_*H*_ − *β*_*H*_ and *β* ≔ *β*_*L*_ − *α*_*L*_, we can simplify the expressions of consumers’ utilities obtained from GB. Precisely, consumers in *H* and *L* segments pay the price *p*_*G*_ through GB and obtain the net utilities equal to *v*_*H*_ − *p*_*G*_ − *αq*_*G*_ and *v*_*L*_ − *p*_*G*_ + *βq*_*G*_, respectively. Of course, only those whose net utility exceeds 0 will participate in GB. Considering that the marginal influence of network effect is much less than the basic valuation for the product from the perspective of consumers, we assume *b* >> *α*, *β* throughout this paper.

Obviously, consumers in *H* segment are more individualistic while consumers in *L* segment are more collectivistic. If there are only individualistic consumers, i.e., *m* = 1, the seller’s optimal strategy is to sell only to individuals through IB and the seller’s revenue is a convex function of IB price. In this case, the optimal IB price is equal to *p*_*I*_ = (*b* + *c*)/2 which results in an individual purchasing quantity equal to *q*_*I*_ = (*b* + *c*)/(2*b*). In contrast, if there are only collectivistic consumers, i.e., *m* = 0, the seller’s optimal strategy is to offer only GB option and the seller’s revenue is also a convex function of GB price. In this case, the optimal GB price is derived equal to *p*_*G*_ = *b*/2 resulting in a group purchasing quantity of *q*_*G*_ = *b*/(2*b* − 2*β*). However, when *m* ∈ (0,1), the optimal pricing strategy is not straightforward. The seller has to maximize revenue with respect to the group buying price and the corresponding threshold quantity through GB, and the individual price through IB.

## 3 Optimal pricing strategies

In this section, we will analyze the optimal prices and revenues for the seller if it offers individual buying (IB), group buying (GB), and buying through both mechanisms (MIX). Some useful insights are also derived by comparative analysis in this section.

### 3.1 Offering only individual buying (IB)

If the seller only offers IB, there will be no network effect for the consumers. Given the price *p*_*I*_ through IB, consumers in *H* and *L* segments obtain the net utilities equal to *v*_*H*_ − *p*_*I*_ and *v*_*L*_ − *p*_*I*_, respectively. Considering the distribution of consumers’ valuations, the feasible price under the IB mechanism should satisfy 0 < *p*_*I*_ < *b* + *c*, otherwise there will be no interested consumers. Assuming that consumers with net utilities no less than 0 will buy the product, given 0 < *p*_*I*_ ≤ *b*, the demands of *H* and *L* segments can be expressed as $q_{IH}=m(1-\frac{p_{I}-c}{b})$ and $q_{IL}=(1-m)(1-\frac{p_{I}}{b})$, respectively. Thus, the seller’s revenue can be expressed as follows:

$$R_{I}=p_{I}[m(1-\frac{p_{I}-c}{b})+(1-m)(1-\frac{p_{I}}{b})]$$ (1)

The optimal pricing strategy is determined by solving the first-order condition with respect to price, i.e., *dR*_*I*_/*dp*_*I*_ = 0. Therefore, the feasible optimal price and corresponding revenue in this case are equal to $p_{I}^{*}=(b+cm)/2$ and $R_{I}^{*}=(b+cm{)}^{2}/\left(4b\right)$, respectively. Note that 0 < *c* < *b* and 0 < *m* < 1, and thus $0<p_{I}^{*}\leq b$, which means that the expression (1) about the seller’s profit maximization as well as the solution is valid.

If *b* ≤ *p*_*I*_ < *b* + *c*, the demand of *H* segment can be still expressed as $q_{IH}=m(1-\frac{p_{I}-c}{b})$, but there will be no consumer in *L* segment buying the product. Thus, the seller’s revenue can be expressed as follows:

$$R_{I}=p_{I}m(1-\frac{p_{I}-c}{b})$$ (2)

Obviously, the feasible optimal price and corresponding revenue in this case are equal to *p*_*I*_ = *b* and *R*_*I*_ = *cm*, respectively. Since (*b* + *cm*)^2^/(4*b*) ≥ *cm*, considering both the cases, we conclude that the optimal IB price and maximum revenue are equal to $p_{I}^{*}=(b+cm)/2$ and $R_{I}^{*}=(b+cm{)}^{2}/\left(4b\right)$, respectively.

Confirming intuition, individual buying revenue ($R_{I}^{*}$) increases with the proportion of individualistic consumers (*m*) and the difference of valuations between the two segments (*c*). Employing the assumption of 0 < *c* < *b*, we find that $R_{I}^{*}$ also increases with *b*, i.e., $dR_{I}^{*}/db>0$, wherein *b* captures the average valuation of all consumers in some extent.

### 3.2 Offering only group buying (GB)

If the seller only offers GB, consumers in both segments will perceive positive and negative network effects. Given the price *p*_*G*_ through GB, consumers in *H* and *L* segments obtain the net utilities equal to *v*_*H*_ − *p*_*G*_ − *αq*_*G*_ and *v*_*L*_ − *p*_*G*_ + *βq*_*G*_, respectively. Thus, given *p*_*G*_ + *αq*_*G*_ ≤ *b* + *c* and *p*_*G*_ − *βq*_*G*_ ≤ *b*, the demands of *H* and *L* segments can be expressed as $q_{GH}=m(1-\frac{p_{G}+\alpha q_{G}-c}{b})$ and $q_{GL}=(1-m)(1-\frac{p_{G}-\beta q_{G}}{b})$, respectively. For the seller, the threshold quantity adopting GB should be set equal to *q*_*G*_ = *q*_*GH*_ + *q*_*GL*_, and thus we can derive $q_{G}=\frac{b+cm-p_{G}}{b+m\alpha -(1-m)\beta }$. Therefore, with the restrictions of *p*_*G*_ + *αq*_*G*_ ≤ *b* + *c* and *p*_*G*_ − *βq*_*G*_ ≤ *b*, the seller’s revenue offering only GB can be expressed as follows:

$$R_{G}=p_{G}q_{G}=\frac{p_{G}(b+cm-p_{G})}{b+m\alpha -(1-m)\beta }$$ (3)

The optimal pricing strategy is determined by solving the first-order condition with respect to price, i.e., *dR*_*G*_/*dp*_*G*_ = 0. Thus, the feasible optimal price and corresponding threshold quantity are equal to $p_{G}^{*}=\frac{b+cm}{2}$ and $q_{G}^{*}=\frac{b+cm}{2[b+m\alpha -(1-m)\beta ]}$, respectively. And the resulting revenue is $R_{G}^{*}=\frac{{\left(b+cm\right)}^{2}}{4[b+m\alpha -(1-m)\beta ]}$. Note that 0 < *c* < *b*, 0 ≤ *m* ≤ 1, and *b* >> *α*, *β*, and thus $p_{G}^{*}+\alpha q_{G}^{*}\leq b+c$ and $p_{G}^{*}-\beta q_{G}^{*}\leq b$, which means that the expression (3) about the seller’s profit maximization as well as the solution is valid.

Next, we will discuss the other two cases. First, for the case with *p*_*G*_ − *βq*_*G*_ ≥ *b*, there will be no consumer in *L* segment buying the product through GB. Since consumers in *H* segment are more sensitive to the negative network effect, the maximal revenue of GB strategy in this case is obviously less than that of IB strategy for the seller. Therefore, to focus on the comparation between sales strategies, we do not further consider this case.

Second, for the case with *p*_*G*_ + *αq*_*G*_ ≥ *b* + *c* and *p*_*G*_ − *βq*_*G*_ ≤ *b*, there will only be consumers in *L* segment buying the product through GB. In this case, the threshold quantity adopting GB should be set equal to $q_{G}=q_{GL}\text{=}(1-m)(1-\frac{p_{G}-\beta q_{G}}{b})$, and thus we can derive $q_{G}=\frac{\left(1-m)(b-p_{G}\right)}{b-(1-m)\beta }$. For this case, the seller’s revenue offering only GB can be expressed as follows:

$$R_{G}=p_{G}q_{G}=\frac{(1-m)(b-p_{G})p_{G}}{b-(1-m)\beta }$$ (4)

Obviously, this function (4) is convex with *p*_*G*_ and gets its maximum value at *p*_*G*_ = *b*/2. However, when *p*_*G*_ = *b*/2 we have $q_{G}=\frac{(1-m)b/2}{b-(1-m)\beta }$, and thus *p*_*G*_ + *αq*_*G*_ < *b* + *c* due to *b* >> *α*, *β* and 0 < *m* < 1, which means that the seller’s revenue maximization problem in this case will degenerate into the case with restrictions of *p*_*G*_ + *αq*_*G*_ ≤ *b* + *c* and *p*_*G*_ − *βq*_*G*_ ≤ *b*, i.e. problem (3). Based on the above analysis, we conclude that the optimal GB price and maximum revenue are equal to $p_{G}^{*}=\frac{b+cm}{2}$ and $R_{G}^{*}=\frac{{\left(b+cm\right)}^{2}}{4[b+m\alpha -(1-m)\beta ]}$, respectively. And the corresponding threshold quantity is equal to $q_{G}^{*}=\frac{b+cm}{2[b+m\alpha -(1-m)\beta ]}$.

Comparing the maximum revenue $R_{G}^{*}$ under GB mechanism with $R_{I}^{*}$ under IB mechanism, we have that $R_{G}^{*}-R_{I}^{*}=\frac{{\left(b+cm\right)}^{2}}{4}(\frac{1}{b+m\alpha -(1-m)\beta }-\frac{1}{b})$. It is obvious that $R_{G}^{*}>R_{I}^{*}$ when *mα* < (1−*m*)*β*, i.e., GB strategy dominates IB strategy as stated in the following proposition.

**Proposition 1**. *For the market*, *if mα* < (1−*m*)*β*, *it may result in more revenue offering only GB than offering only IB*. *Otherwise*, *IB strategy gains no less revenue than GB strategy*.

Proposition 1 implies that either a larger proportion of *L* segment (1 − *m*) or a more significant positive network effect (*β*) can make GB being more attractive than IB. Contrarily, when the positive network effect is relatively low, i.e., *β* << *α*, the seller should choose to sell product only through IB. These findings are more useful for practice. Suppose that a firm is planning to sell a unique new product to the market. Because the market is unfamiliar with the product, consumers are more likely to follow group decisions and their utilities can usually be enhanced by the presence of other buyers. In this case, almost all consumers are of *L* segment with a high positive network effect, which implies *mα* < (1 − *m*)*β*, and thus GB undoubtedly dominates IB for the firm according to Proposition 1. However, if the product is an “old” one, more consumers identify highly with the product. Even for those whose valuations for the product are low, the negative network effect is more significant than the positive network effect buying with others. In this case, it is more likely that *mα* > (1 − *m*)*β*, which implies that the firm should choose IB rather than GB according to Proposition 1.

Besides, we also get some useful findings from the values of optimal prices and purchasing quantities as shown in Proposition 2.

**Proposition 2**. *For the seller*, *the optimal price offering only IB equals the optimal price offering only GB*. *And for the market*, *if mα* < (1−*m*)*β*, *the optimal GB strategy can expand demand compared to IB strategy*.

The first part of Proposition 2 is interesting, which seems to be inconsistent with common sense. The reason is that there is only one option, GB or IB, offered for the market. From the next section, we will find that the optimal prices are different when both options are offered. Another explanation is that GB brings negative network effect to *H* segment while positive network effect to *L* segment. As a result, given the same price, GB will realize a demand of fewer consumers in *H* segment and more consumers in *L* segment compared to IB. This kind of twofold influence makes the optimal price of IB still maintain the optimality in GB. However, the purchasing quantities may be different. Given $p_{I}^{*}=p_{G}^{*}=\frac{b+cm}{2}$, the demands of consumers in *H* segment under IB and under GB equal $q_{IH}=\frac{m}{2b}(b+2c-cm)$ and $q_{GH}=\frac{m}{2b}[b+2c-cm-\frac{\alpha (b+cm)}{b+m\alpha -(1-m)\beta }]$, respectively. Normally, the marginal influence of network effect is much less than the basic valuation for the product from the perspective of consumers, i.e., *b* >>*α*, *β*, and thus *q*_*IH*_ > *q*_*GH*_. For *L* segment, the demands of consumers under IB and under GB equal $q_{IL}=\frac{1-m}{2b}(b-cm)$ and $q_{GL}=\frac{1-m}{2b}[b-cm+\frac{\beta (b+cm)}{b+m\alpha -(1-m)\beta }]$, respectively. For the same reason of *b* >> *α*, *β*, we have *q*_*IL*_ < *q*_*GL*_. That is, GB increases the demand of *L* segment while decreases the demand of *H* segment compared to IB. Considering the total demand, we have $q_{I}=q_{IH}+q_{IL}=\frac{b+cm}{2b}$ and $q_{G}=q_{GH}+q_{GL}=\frac{b+cm}{2b+2[m\alpha -(1-m)\beta ]}$ respectively for IB strategy and GB strategy. It is obvious that *q*_*G*_ > *q*_*I*_ when *mα* < (1 − *m*)*β*, which is stated by the second part of Proposition 2.

From Proposition 1 and Proposition 2, we may propose a dynamic pricing strategy for a firm who is planning to sell a unique product to a new market. At the beginning, because the market is unfamiliar with the product and the positive network effect is more significant, the firm should offer only GB option to enhance consumers’ valuation for the product and to expand the market quickly. With the improving of consumers’ valuation and identification, the negative network effect becomes more significant and thus the market moves to a mature stage. At this time, the firm should offer IB option rather than GB option to skim more revenue. Of course, offering both IB option and GB option (MIX strategy) might result in a much more revenue during certain sales period, which is the focus of the next section.

### 3.3 Offering both IB and GB (MIX)

As mentioned in the previous section, the seller may adopt a MIX selling strategy, i.e., offering IB option and GB option simultaneously, to improve revenue. To avoid confusion, let *p*_*MI*_ be the unit price and *q*_*MI*_ be the quantity sold through IB under a MIX strategy. Let *q*_*MG*_ be the threshold quantity adopting GB at unit price *p*_*MG*_ under a MIX strategy. The seller’s objective is to maximize revenue with respect to the possible values of *p*_*MI*_, *p*_*MG*_, and *q*_*MG*_, under MIX strategy.

For consumers in *H* segment, the net utilities obtained from IB and GB under MIX strategy are equal to *v*_*H*_ − *p*_*MI*_ and *v*_*H*_ − *p*_*MG*_ − *αq*_*MG*_, respectively. Correspondingly, consumers in *L* segment obtain the net utilities from IB and GB equal to *v*_*L*_ − *p*_*MI*_ and *v*_*L*_ − *p*_*MG*_ + *βq*_*MG*_, respectively. Obviously, the difference between *p*_*MI*_ and *p*_*MG*_ should not be too large for a feasible MIX strategy. Otherwise, if *p*_*MI*_ − *p*_*MG*_ > *αq*_*MG*_, consumers in both *H* segment and *L* segment cannot choose IB option because it results in a less net utility than GB option. In this case, the MIX strategy will degenerate into a pure GB strategy. Therefore, offering any MIX strategy with *p*_*MI*_ − *p*_*MG*_ > *αq*_*MG*_ is not better than offering an optimal pure GB strategy for the seller. On the other hand, if *p*_*MI*_ − *p*_*MG*_ <−*βq*_*MG*_, all consumers will favor IB option because it results in a more net utility than GB option, which means that offering any MIX strategy with *p*_*MI*_ − *p*_*MG*_ <−*βq*_*MG*_ is not better than offering an optimal pure IB strategy for the seller. Specifically, if *p*_*MI*_ − *p*_*MG*_ = *αq*_*MG*_ (or *p*_*MI*_ − *p*_*MG*_ = −*βq*_*MG*_), it is indifferent between IB option and GB option for consumers in *H* segment (or *L* segment), we assume they favor IB option. Summarily, we first derive the following Proposition 3 which describes the feasible condition for a MIX strategy.

**Proposition 3**. *For the market*, *any feasible MIX strategy should satisfy the condition of* −*βq*_*MG*_ < *p*_*MI*_ − *p*_*MG*_ ≤ *αq*_*MG*_ *under which all consumers in H segment favor IB option while all consumers in L segment favor GB option*.

Given *p*_*MI*_, *p*_*MG*_, and *q*_*MG*_, we firstly suppose that the feasible condition of −*βq*_*MG*_ < *p*_*MI*_ − *p*_*MG*_ ≤ *αq*_*MG*_ has been satisfied. Under this condition, all consumers in *H* segment favor IB option and those with a net utility *v*_*H*_ − *p*_*MI*_ ≥ 0 will purchase the product through IB option. Contrarily, all consumers in *L* segment favor GB option and those with a net utility *v*_*L*_ − *p*_*MG*_ + *βq*_*MG*_ ≥ 0 will purchase the product through GB option. Thus, two segments will realize demands equal to $q_{MI}=m(1-\frac{p_{MI}-c}{b})$ and $q_{MG}=\frac{\left(1-m)(b-p_{MG}\right)}{b-(1-m)\beta }$, respectively. Therefore, the seller’s revenue offering MIX strategy can be expressed as follows:

$$R_{M}=p_{MI}m(1-\frac{p_{MI}-c}{b})+\frac{p_{MG}(1-m)(b-p_{MG})}{b-(1-m)\beta }$$ (5)

The optimal pricing strategy is also determined by solving the first-order conditions with respect to prices, i.e., *∂R*_*M*_ /*∂p*_*MI*_ = 0 and *∂R*_*M*_ /*∂p*_*MG*_ = 0. As a result, the optimal GB price and the corresponding threshold quantity for GB option are derived equal to $p_{MG}^{*}=b/2$ and $q_{MG}^{*}=\frac{b(1-m)}{2[b-(1-m)\beta ]}$, respectively. And the optimal IB price is equal to $p_{MI}^{*}=\left(b+c\right)/2$ which results in an individual purchasing demand of $q_{MI}=\frac{m(b+c)}{2b}$. From the last part of Section 1, we have known that *P*_*I*_ = (*b* + *c*)/2 and *p*_*G*_ = *b*/2 are both the best prices resulting in the maximum revenues for the seller facing each segment separately, which means that the proposed MIX strategy with $p_{MI}^{*}=\left(b+c\right)/2$ and $p_{MG}^{*}=b/2$ undoubtedly dominates any pure option of IB or GB. Remember that we need the condition of −*βq*_*MG*_ < *p*_*MI*_ − *p*_*MG*_ ≤ *αq*_*MG*_ to guarantee the validity of this solution. That is, it has to satisfy $\frac{c}{2}\leq \frac{\alpha b(1-m)}{2[b-(1-m)\beta ]}$, and thus we have the following Proposition 4.

**Proposition 4**. *For the market, if $1-m\geq \frac{bc}{b\alpha +c\beta }$, the optimal MIX strategy with IB price equal to $p_{MI}^{*}=\left(b+c\right)/2$, GB price equal to $p_{MG}^{*}=b/2$, and threshold quantity for GB equal to $q_{MG}^{*}=\frac{b(1-m)}{2[b-(1-m)\beta ]}$, dominates any pure strategy of IB or GB*.

From the above analysis and Proposition 3, we have known that consumers in *H* segment will favor IB and consumers in *L* segment will favor GB under an optimal MIX strategy. Thus, we can deduce that the IB price *p*_*MI*_ and the GB price *p*_*MG*_ under an optimal MIX strategy should satisfy *p*_*MG*_ ≤ *p*_*MI*_. Otherwise, if −*βq*_*MG*_ < *p*_*MI*_ − *p*_*MG*_ < 0 under a feasible MIX strategy, the seller can improve revenue by increasing *p*_*MI*_ to *p*_*MG*_ when *p*_*MG*_ ≤ (*b* + *c*)/2 or by modifying both *p*_*MI*_ and *p*_*MG*_ to (*b* + *c*)/2 when *p*_*MG*_ > (*b* + *c*)/2 without changing the feasible condition of −*βq*_*MG*_ < *p*_*MI*_ − *p*_*MG*_ ≤ *αq*_*MG*_. Based on this necessary condition of *p*_*MG*_ ≤ *p*_*MI*_, we can further deduce that the prices *p*_*MI*_ and *p*_*MG*_ under an optimal MIX strategy should satisfy *b*/2 ≤ *p*_*MG*_ ≤ *p*_*MI*_ ≤ (*b* + *c*)/2 when $1-m<\frac{bc}{b\alpha +c\beta }$. Otherwise, if $p_{MG}\leq p_{MI}<\frac{b}{2}$ (or $p_{MG}<\frac{b}{2}\leq p_{MI}\leq \frac{b+c}{2}$), the seller can improve revenue by increasing both *p*_*MG*_ and *p*_*MI*_ (or only *p*_*MG*_) to *b*/2. If $\frac{b+c}{2}<p_{MG}\leq p_{MI}$ (or $\frac{b}{2}\leq p_{MG}\leq \frac{b+c}{2}<p_{MI}$), the seller can improve revenue by decreasing both *p*_*MI*_ and *p*_*MG*_ (or only *p*_*MI*_) to (*b* + *c*)/2. If *p*_*MG*_ < *b*/2 and *p*_*MI*_ > (*b* + *c*)/2, the seller can improve revenue by simultaneously increasing *p*_*MG*_ to *b*/2 and decreasing *p*_*MI*_ to (*b* + *c*)/2. Note that the seller needs to set a new threshold quantity *q*_*MG*_ satisfying the feasible condition of *p*_*MI*_ − *p*_*MG*_ ≤ *αq*_*MG*_ for above price adjustments. In the same manner, the seller can continuously increase *p*_*MI*_ or/and decrease *p*_*MG*_ to obtain a much more revenue until *p*_*MI*_ − *p*_*MG*_ = *αq*_*MG*_. Summarily, we have the following Proposition 5 describing the necessary conditions for an optimal MIX strategy when $1-m<\frac{bc}{b\alpha +c\beta }$.

**Proposition 5**. *For the market, if $1-m<\frac{bc}{b\alpha +c\beta }$, the IB price p*_*MI*_, *the GB price p*_*MG*_, *and the threshold quantity q*_*MG*_ *for GB option under an optimal MIX strategy should satisfy b*/2 ≤ *p*_*MG*_ < *p*_*MI*_ ≤ (*b* + *c*)/2 *and p*_*MI*_ − *p*_*MG*_ = *αq*_*MG*_.

Given the IB price *p*_*MI*_ and the GB price *p*_*MG*_ under an optimal MIX strategy, two segments will realize demands respectively equal to $q_{MI}=m(1-\frac{p_{MI}-c}{b})$ and $q_{MG}=\frac{\left(1-m)(b-p_{MG}\right)}{b-(1-m)\beta }$ from Proposition 3. Based on Proposition 5, we have that *p*_*MI*_ = *p*_*MG*_ + *αq*_*MG*_ for an optimal MIX strategy, i.e., $p_{MI}\text{=}p_{MG}+\frac{\alpha (1-m)(b-p_{MG})}{b-(1-m)\beta }$. Thus, let $A:=\frac{\alpha (1-m)}{b-(1-m)\beta }$, the seller’s revenue offering MIX strategy when $1-m<\frac{bc}{b\alpha +c\beta }$ can be expressed as follows:

$$R_{M}=m[p_{MG}+A(b-p_{MG})][1-\frac{p_{MG}+A(b-p_{MG})-c}{b}]+\frac{Ap_{MG}(b-p_{MG})}{\alpha }$$ (6)

Solving the first-order condition with respect to price, i.e., *dR*_*M*_/*dp*_*MG*_ = 0, we can derive the optimal GB price equal to $p_{MG}^{*}=\frac{Ab^{2}+m\alpha (A-1)(2Ab-b-c)}{2Ab+2m\alpha {\left(A-1\right)}^{2}}$. Correspondingly, the threshold quantity for GB and the optimal IB price are equal to $q_{MG}^{*}=\frac{A^{2}b^{2}+Am\alpha (A-1)(c-b)}{2\alpha [Ab+m\alpha {\left(A-1\right)}^{2}]}$ and $p_{MI}^{*}=\frac{Ab^{2}(A+1)+m\alpha {\left(A-1\right)}^{2}(b+c)}{2Ab+2m\alpha {\left(A-1\right)}^{2}}$, respectively. Under the proposed optimal MIX strategy, there will be $q_{MG}^{*}$ consumers in *L* segment buying product through GB option and $q_{MI}=\frac{Abm(b+2c-Ab)+m^{2}\alpha (b+c){\left(A-1\right)}^{2}}{2b[Ab+m\alpha {\left(A-1\right)}^{2}]}$ consumers in *H* segment buying product through IB option. And thus, the maximum revenue offering an optimal MIX strategy is equal to $R_{M}^{*}=p_{MI}^{*}q_{MI}+p_{MG}^{*}q_{MG}^{*}$.

Compared to IB strategy, we find that offering MIX strategy can always improve the seller’s revenue when $1-m<\frac{bc}{b\alpha +c\beta }$. First, from the analysis of above paragraph, we have that $\frac{b}{2}<p_{MG}^{*}<p_{MI}^{*}<\frac{b+c}{2}$ and there certainly exists another feasible MIX strategy with $\frac{b}{2}<p_{MG}^{o}<\frac{b+cm}{2}<p_{MI}^{o}<\frac{b+c}{2}$ whose resulting revenue is less than the proposed optimal MIX strategy with $p_{MI}^{*}$ and $p_{MG}^{*}$. Because the positive network effect is significant for consumers in *L* segment, offering only GB with $p_{G}=\frac{b+cm}{2}$ will obtain more revenue from *L* segment than offering only IB with $p_{I}=\frac{b+cm}{2}$. Again from the last paragraph of Section 2, we have known that the revenue offering only GB to *L* segment is decreasing with *p*_*G*_ when *p*_*G*_ > *b*/2. In a word, offering only IB with $p_{I}=\frac{b+cm}{2}$ obtains less revenue from *L* segment than offering MIX strategy with $p_{MG}^{o}$. On the other hand, offering MIX strategy with $p_{MI}^{o}$ obviously obtains more revenue from *H* segment than offering only IB with $p_{I}=\frac{b+cm}{2}$, because the revenue offering only IB to *H* segment is increasing with *p*_*I*_ when *p*_*I*_ < (*b* + *c*)/2. Note that $p_{I}=\frac{b+cm}{2}$ is the optimal price of offering only IB, and thus we have the following Proposition 6.

**Proposition 6**. *For th*e *market, if $1-m<\frac{bc}{b\alpha +c\beta }$, offering the optimal MIX strategy derived from problem* (6) *dominates offering only IB strategy*.

However, the degree to which offering MIX strategy may dominate offering only GB strategy is not so straightforward. Consider again the proposed feasible MIX strategy with $\frac{b}{2}<p_{MG}^{o}<\frac{b+cm}{2}<p_{MI}^{o}<\frac{b+c}{2}$. Although the proposed MIX strategy obtains more revenue from *H* segment than the optimal GB strategy, the purchasing quantity of *L* segment may be considerably less under MIX strategy than that under GB strategy because of the positive network effect in GB. As a result, the relationship between the respective revenues of MIX strategy and GB strategy depends on actual market parameters. Therefore, the seller can only choose a pricing strategy from offering only GB strategy and offering MIX strategy by comparing the respective revenues as stated in the following Proposition 7.

**Proposition 7**. *For the market, if $1-m<\frac{bc}{b\alpha +c\beta }$, offering only GB strategy dominates offering MIX strategy when $R_{G}^{*}>R_{M}^{*}$, wherein $R_{M}^{*}$ is the maximum revenue derived from problem* (6) *for MIX strategy and $R_{G}^{*}$ is the revenue of an optimal GB strategy derived from problem* (3). *Otherwise*, *offering MIX strategy is dominant*.

## 4 Numerical analyses

In this section we present several numerical examples to illustrate the results of our model as well as some managerial insights. Note that, we normalize the scale of market to 1 in all numerical examples.

### 4.1 Sensitivity analyses under IB

**Fig 1:**
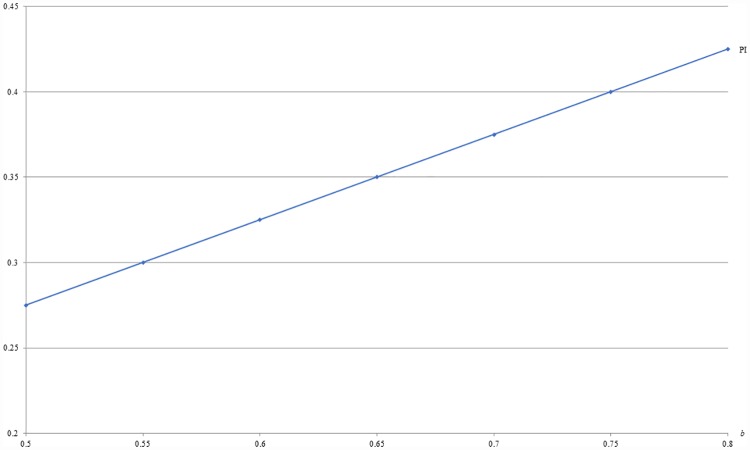
An illustration of the relation between price and *b* under IB.

**Fig 2:**
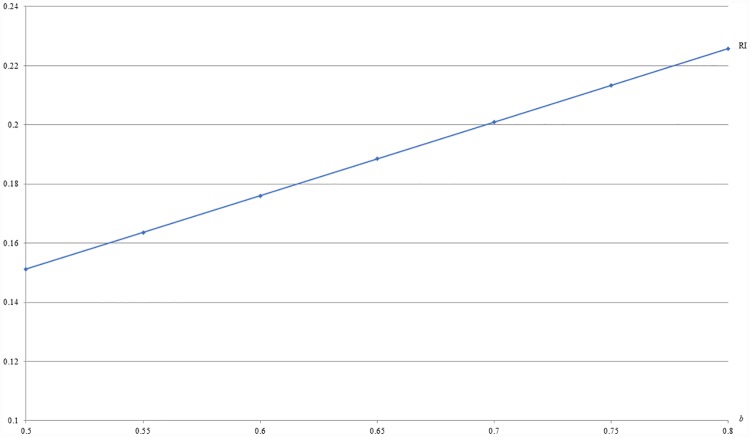
An illustration of the relation between revenue and *b* under IB.

**Example 1**: *m* = 0.5, *c* = 0.1 and 0.5 ≤ *b* ≤ 0.8. For this example, Figs 1 and 2 respectively show that the optimal price (PI) and the resulting revenue (RI) under IB are increasing with *b*, given *m* and *c*.

**Fig 3:**
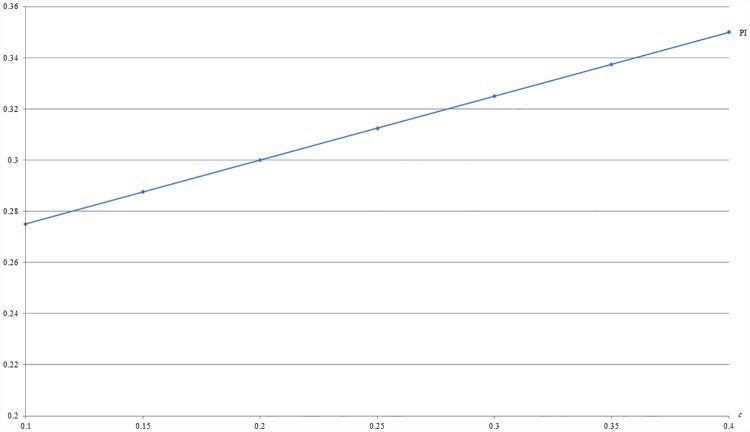
An illustration of the relation between price and *c* under IB.

**Fig 4:**
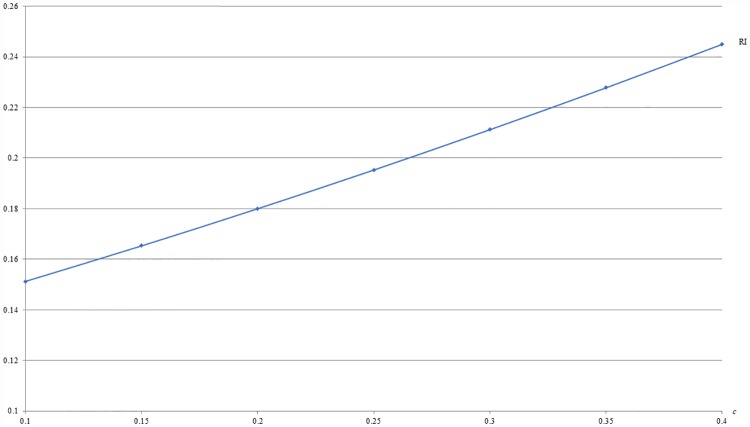
An illustration of the relation between revenue and *c* under IB.

**Example 2**: *m* = 0.5, *b* = 0.5 and 0.1 ≤ *c* ≤ 0.4. For this example, Figs 3 and 4 respectively show that the optimal price (PI) and the resulting revenue (RI) under IB are increasing with the difference of valuations between the two populations, i.e., *c*.

**Fig 5:**
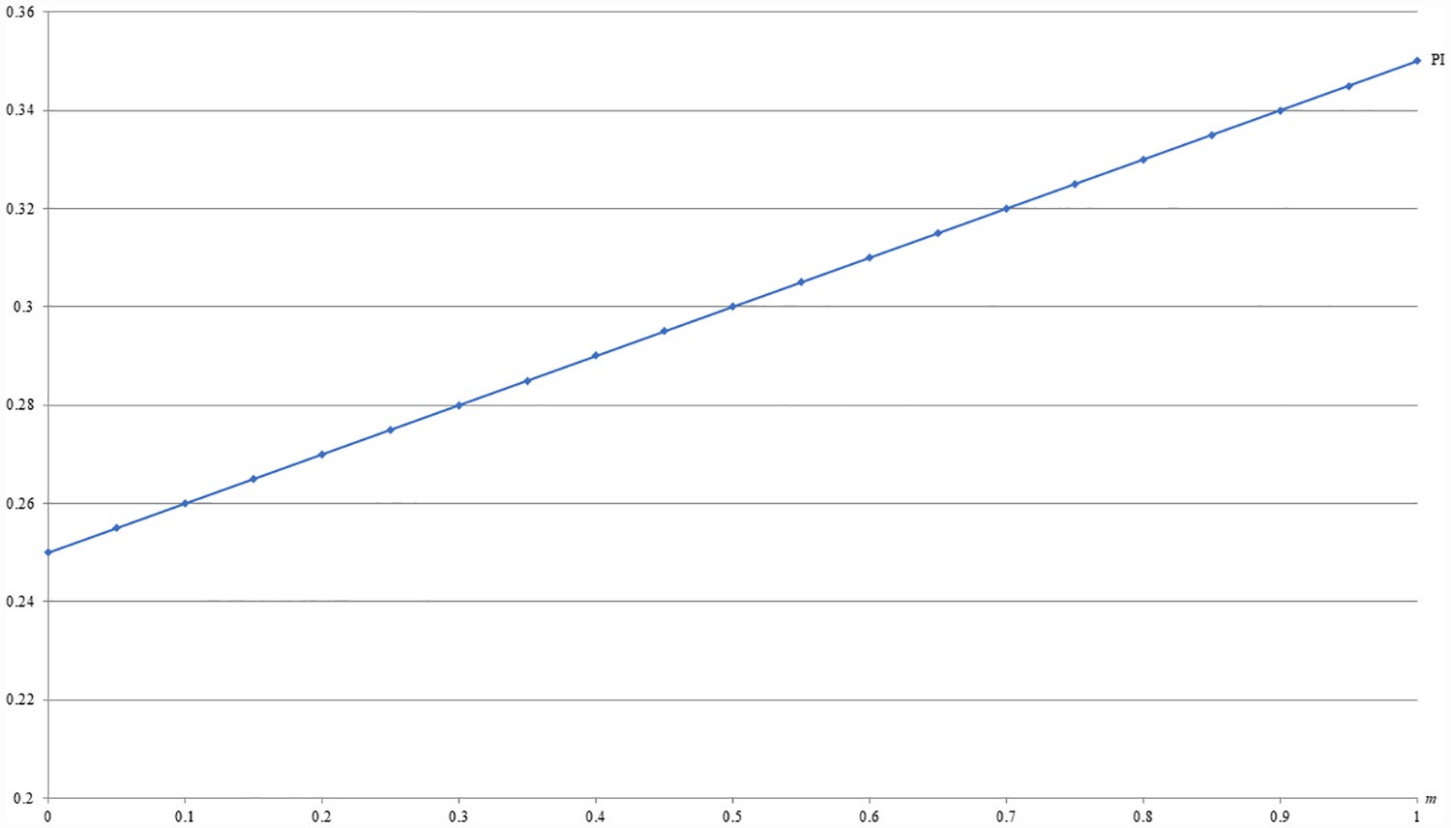
An illustration of the relation between price and *m* under IB.

**Fig 6:**
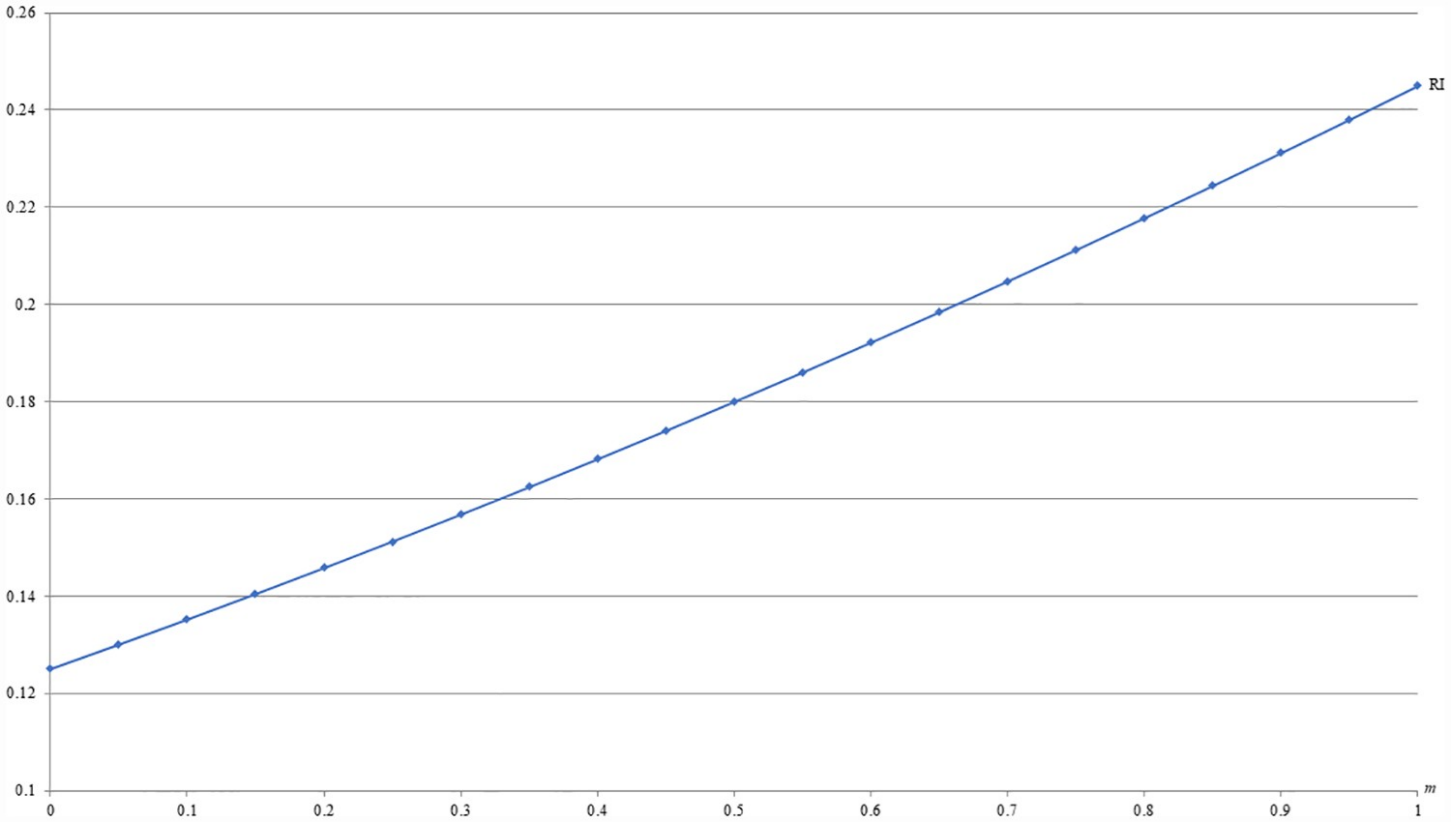
An illustration of the relation between revenue and *m* under IB.

**Example 3**: *b* = 0.5, *c* = 0.2 and 0 ≤ *m* ≤ 1. For this example, Figs 5 and 6 respectively show that the optimal price (PI) and the resulting revenue (RI) under IB are increasing with *m*, given *b* and *c*. That is, the IB strategy is more profitable in the individualistic market than in the collectivistic market, which is in line with the reality.

### 4.2 Comparative analyses between IB and GB

**Fig 7:**
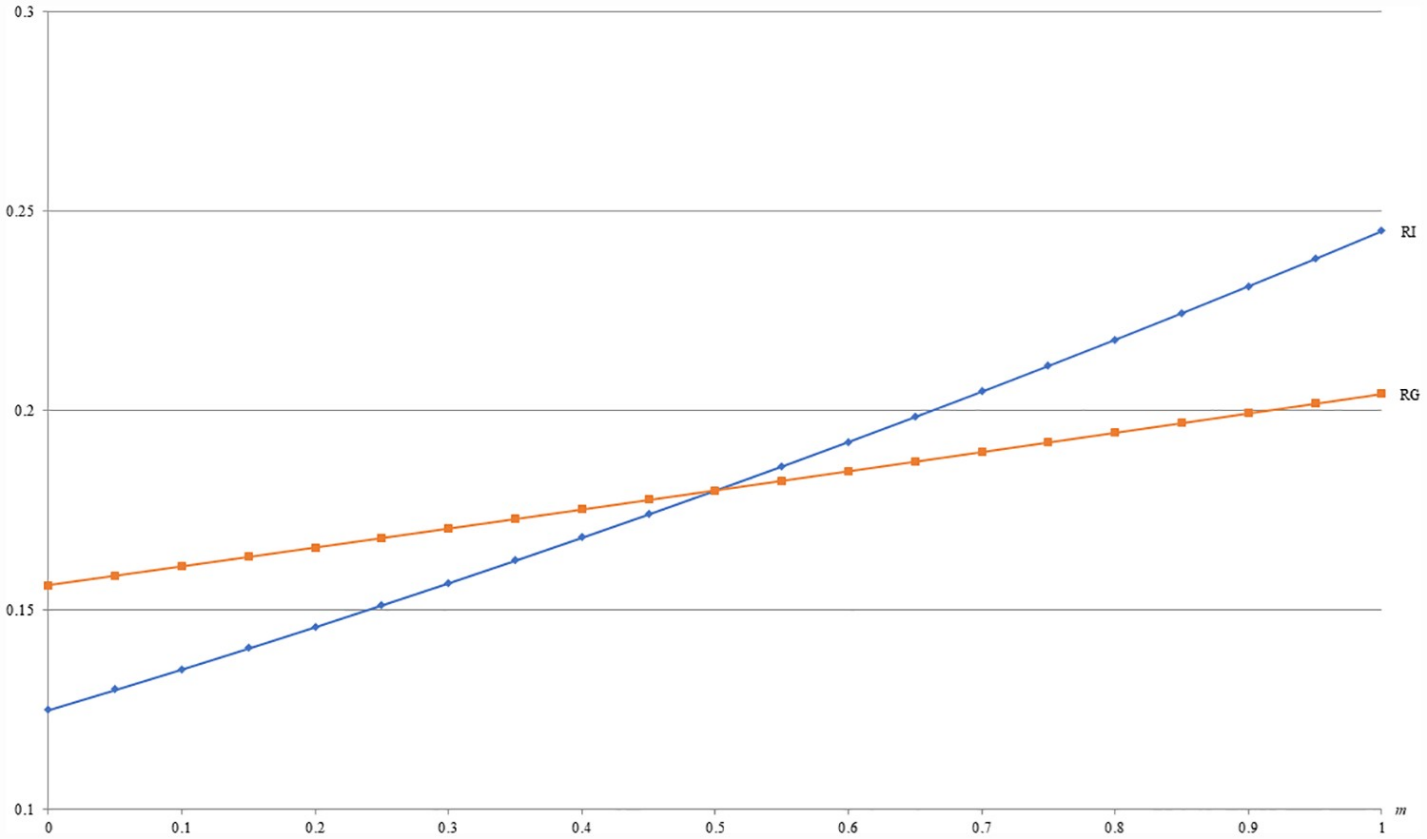
A comparison of revenues between IB and GB with respect to *m*.

**Example 4**: *b* = 0.5, *c* = 0.2, *α* = *β* = 0.1 and 0 ≤ *m* ≤ 1. For this example, Fig 7 shows that the optimal revenue under GB (RG) is no less than that under IB (RI) when 0 ≤ *m* ≤ 0.5 as stated by Proposition 1. This example illustrates that it is more profitable to offer only GB than to offer only IB when the proportion of *H* segment, i.e., *m* is smaller. Otherwise, the seller should offer IB rather than GB.

**Fig 8:**
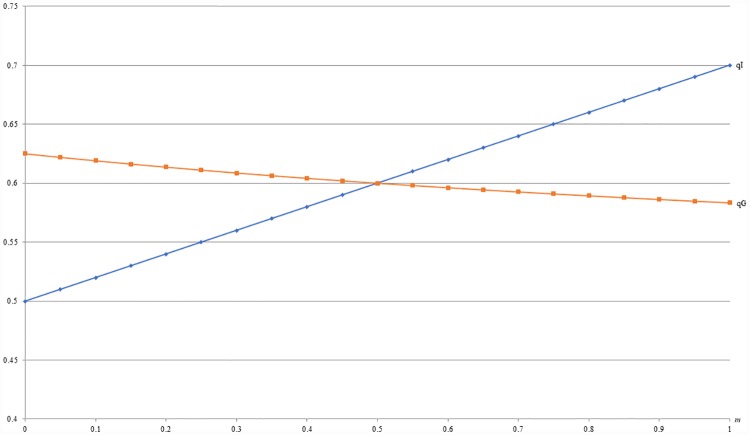
A comparison of demands between IB and GB with respect to *m*.

Besides, for example 4, Fig 8 shows that the resulting demand under GB (qG) is also more than that under IB (qI) when 0 ≤ *m* ≤ 0.5 as stated by Proposition 2. Based on the previous analysis in Section 3.2, this numerical example implies a dynamic pricing strategy for a firm who is planning to sell a unique product to a new market. Generally, the firm should offer only GB option to enhance consumers’ valuation for the product and to expand the market quickly at the beginning when *m* is smaller. As the improving of consumers’ valuation and identification, i.e., *m* becoming larger, the firm should offer IB option rather than GB option to skim more revenue. In fact, many sellers adopt this kind of pricing strategy in real life.

**Fig 9:**
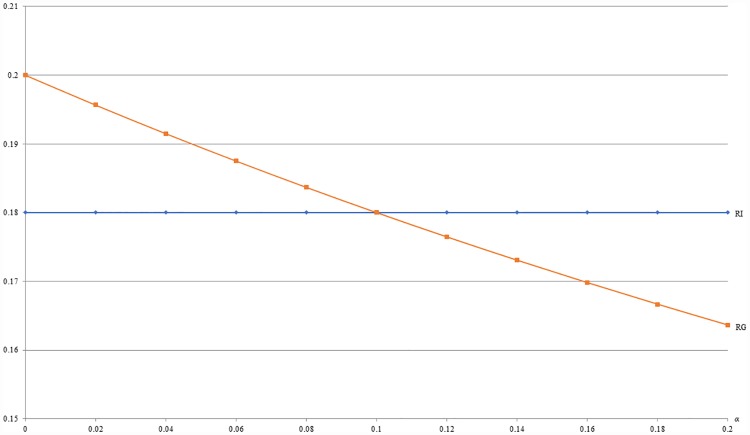
A comparison of revenues between IB and GB with respect to *α*.

**Example 5**: *m* = 0.5, *b* = 0.5, *c* = 0.2, *β* = 0.1 and 0 ≤ *α* ≤ 0.2. Given other market parameters in example 5, Fig 9 shows that the optimal revenue (RG) under GB is decreasing with *α*. As stated by Proposition 1, Fig 9 also shows that the optimal revenue under GB (RG) is no less than that under IB (RI) when *α* ≤ 0.1. Since *α* captures the negative externality of buying with others among *H* segment, this numerical example implies that it might be more profitable to offer IB than GB when consumers in *H* segment are very individualistic.

**Fig 10:**
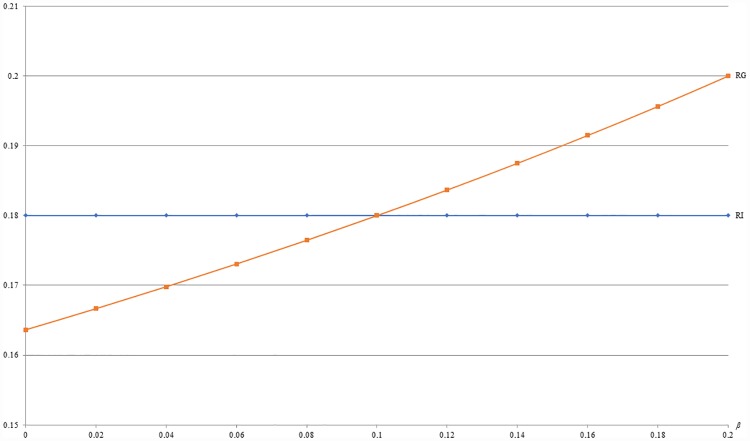
A comparison of revenues between IB and GB with respect to *β*.

**Example 6**: *m* = 0.5, *b* = 0.5, *c* = 0.2, *α* = 0.1 and 0 ≤ *β* ≤ 0.2. For this example, Fig 10 first shows that the optimal revenue (RG) under GB is increasing with *β*. Besides, Fig 10 also shows that the optimal revenue under GB (RG) is no less than that under IB (RI) when *β* ≥ 0.1. Since *β* captures the importance of buying with others among *L* segment, this numerical example implies that it might be more profitable to offer GB than IB when consumers in *L* segment are very collectivistic.

### 4.3 Comparative analyses between IB, GB and MIX

**Fig 11:**
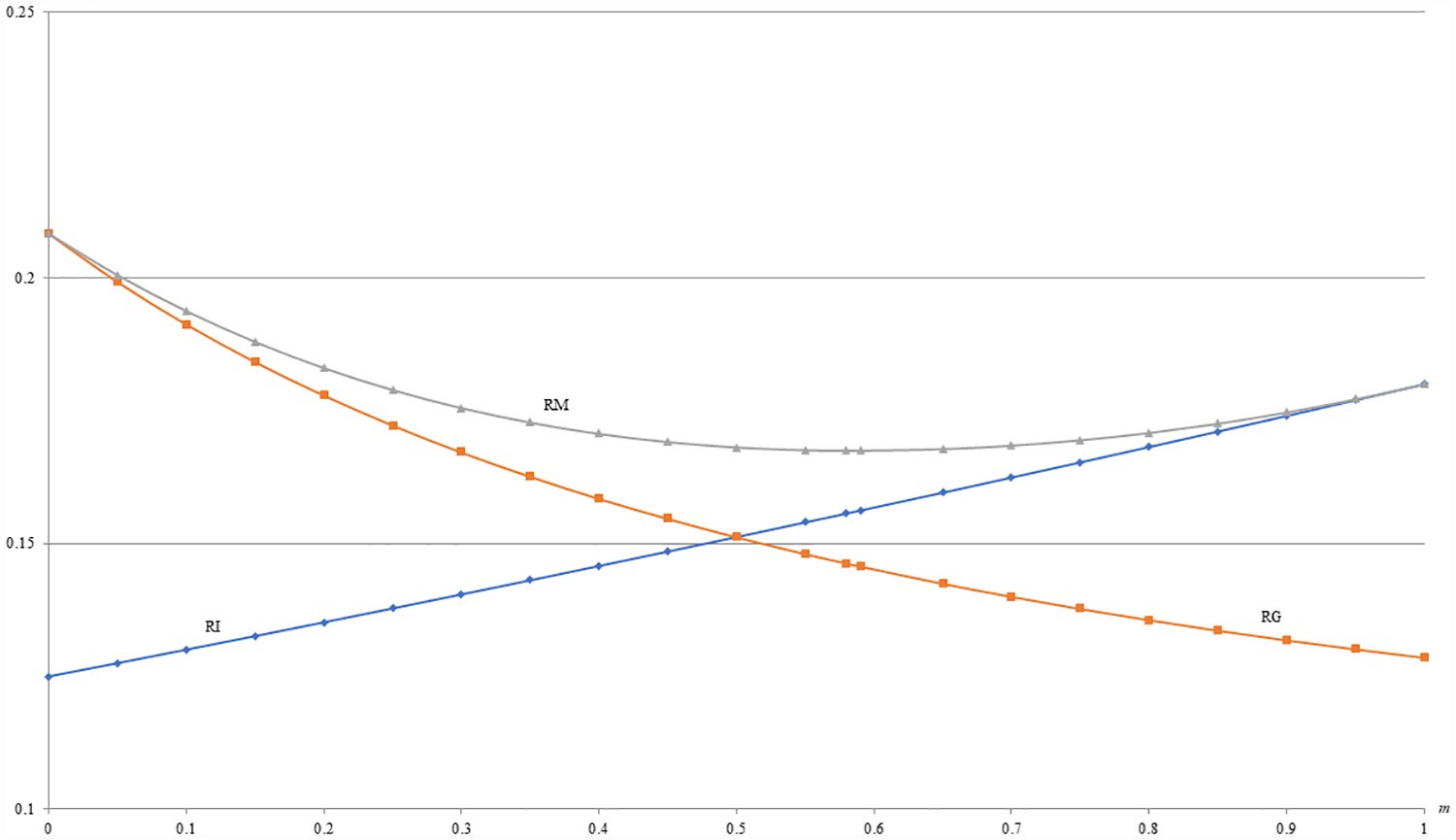
A comparison of revenues between IB, GB and MIX with respect to *m*.

**Example 7**: *b* = 0.5, *c* = 0.1, *α* = *β* = 0.2 and 0 ≤ *m* ≤ 1. For this example, given other market parameters, Fig 11 shows that the optimal revenue under MIX (RM) is first decreasing and then increasing with *m*, i.e., the proportion of *H* segment. Also, Fig 11 shows that the optimal revenue under MIX (RM) is no less than those under both IB (RI) and under GB (RG) as stated by Propositions 4, 6 and 7. Obviously, when there is no *H* segment, i.e., *m* = 0, the MIX strategy will degenerate into a pure GB strategy. By contrast, the MIX strategy will degenerate into a pure IB strategy when *m* = 1.

**Fig 12:**
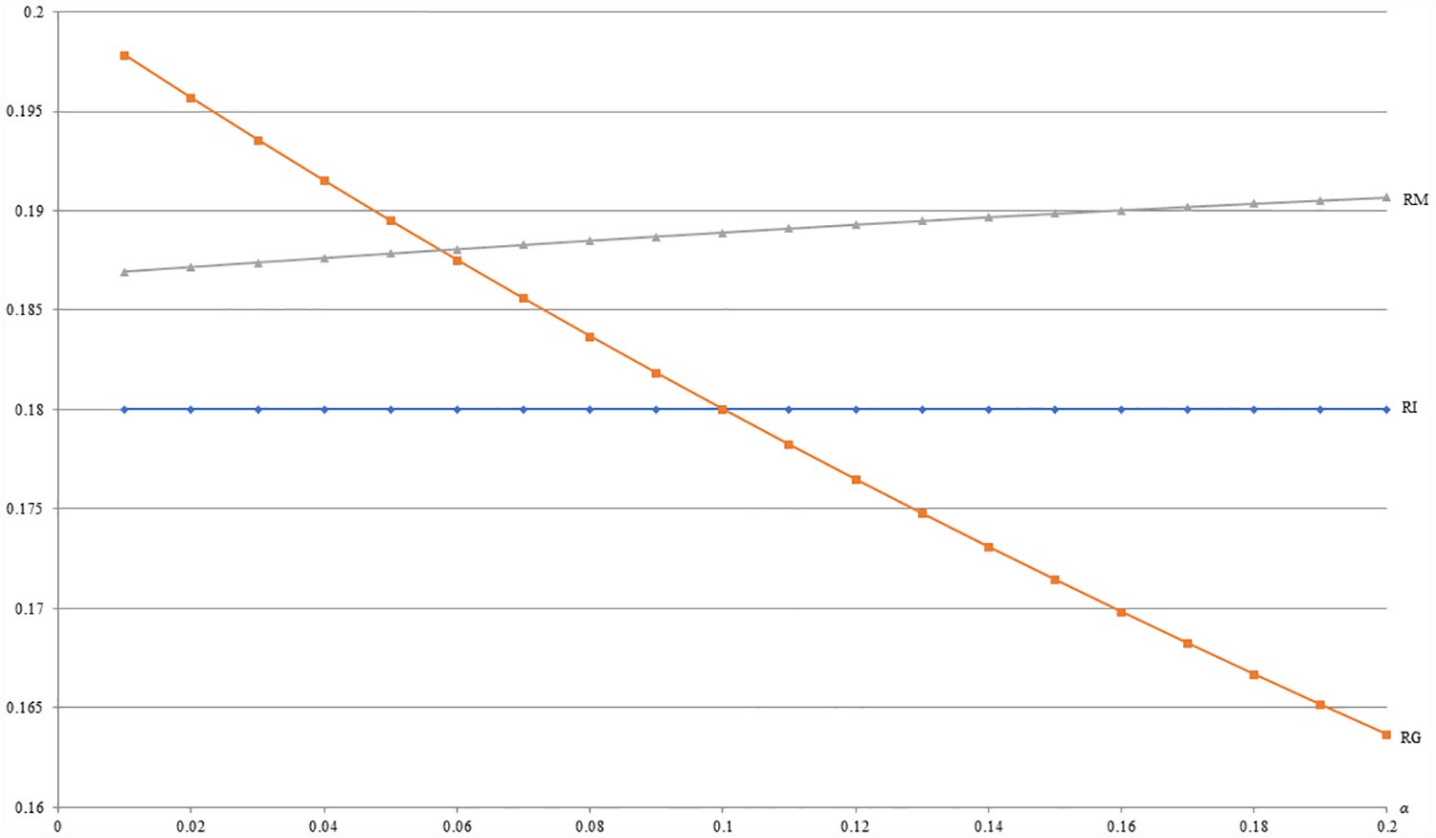
A comparison of revenues between IB, GB and MIX with respect to *α*.

**Example 8**: *m* = 0.5, *b* = 0.5, *c* = 0.2, *β* = 0.1 and 0.01 ≤ *α* ≤ 0.2. For this example, Fig 12 shows that the optimal revenue under MIX (RM) is increasing with *α* and always more than that under IB (RI) as stated by Proposition 6. Besides, Fig 12 also shows that the optimal revenue under MIX (RM) is less than that under GB (RG) when *α* is smaller and more than that under GB (RG) when *α* is larger. As stated by Proposition 7, the seller should choose GB strategy when the negative network effect of *H* segment buying with others is not significant. Otherwise, the MIX strategy is dominant.

**Fig 13:**
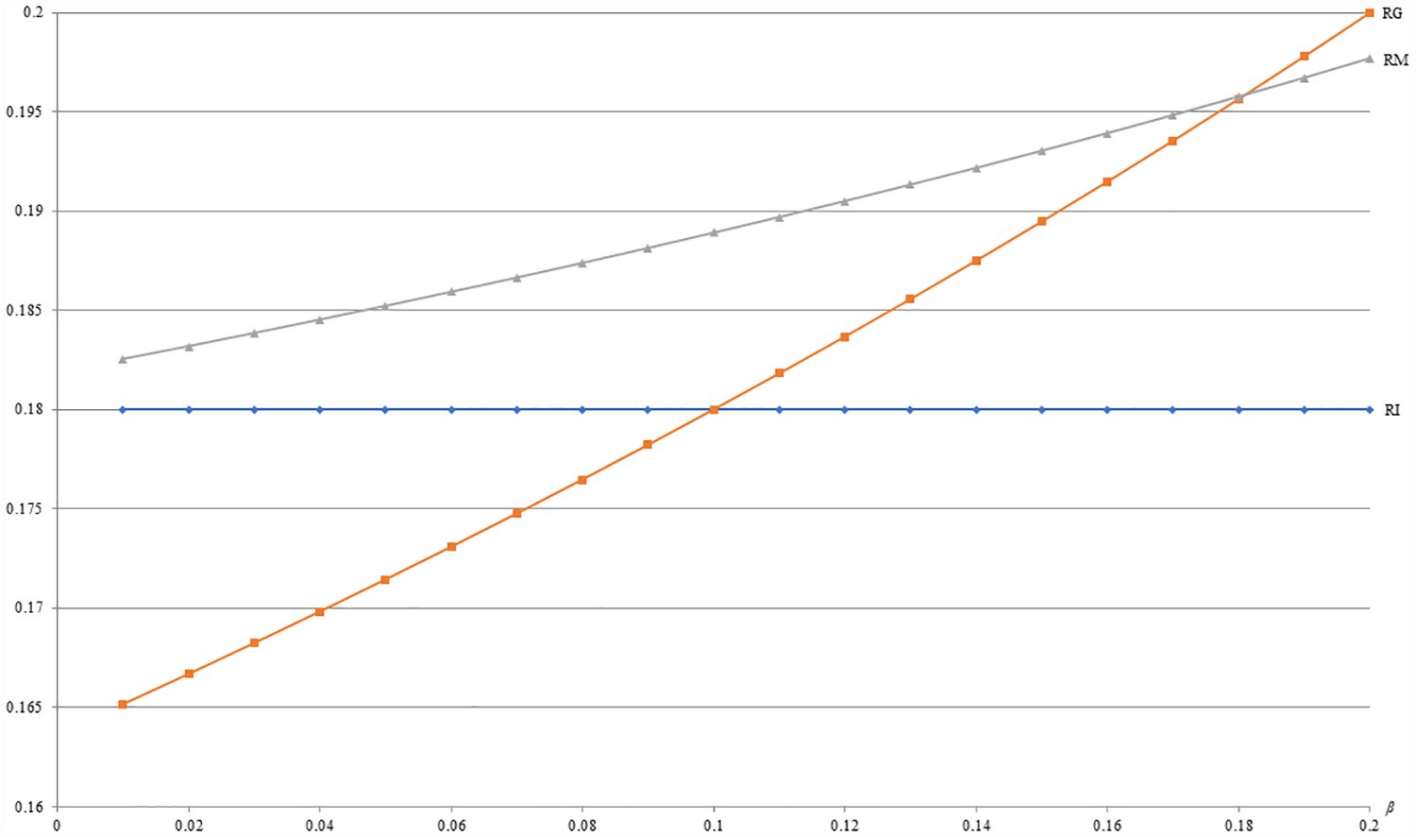
A comparison of revenues between IB, GB and MIX with respect to *β*.

**Example 9**: *m* = 0.5, *b* = 0.5, *c* = 0.2, *α* = 0.1 and 0.01 ≤ *β* ≤ 0.2. For this example, Fig 13 shows that the optimal revenue under MIX (RM) is increasing with *β* and always more than that under IB (RI) as stated by Proposition 6. Also, as stated by Proposition 7, Fig 13 shows that the optimal revenue under MIX (RM) is more than that under GB (RG) when *β* is smaller and less than that under GB (RG) when *β* is larger, which implies that the seller should choose MIX strategy when the positive network effect of *L* segment buying with others is not significant. Otherwise, GB strategy is better than MIX strategy.

## 5 Conclusions

GB is becoming more and more popular in e-commerce. In this paper, we derive the utility consumers obtain from GB by recognizing both positive network effect and negative network effect buying with others. According to their valuations for the product and attitudes to network effects, consumers are classified into two segments. From the perspective of sellers, we compare three possible strategies, i.e., offering only IB option, offering only GB option, and offering both options, and derive the optimal decisions on price and quantity for each strategy. Our result shows that both the network effect and consumer heterogeneity play a significant role in choosing optimal strategy. In particular, offering only GB dominates offering only IB when the positive network effect is sufficiently high or the proportion of low valuation segment is relatively large. We also find that offering both options simultaneously can improve the seller’s revenue compared to offering only IB option, while the seller has to decide offering only GB or offering GB along with IB based on actual market situations.

Several managerial implications can be derived from our study. The positive network effect is quite significant for first-time buyers. Based on this fact, the firm selling a new product can enhance consumers’ valuation and expand the market quickly by offering only GB option. When the product becomes an “old” one, more consumers identify highly with the product and the negative network effect is more significant. In this case, the firm should choose IB strategy (or MIX strategy offering IB and GB simultaneously) rather than GB strategy. Some limitations apply to our model. First, this paper only considers the decision-making problem for a monopoly seller. It is of interest to consider the case under a competitive environment. Second, instead of uniform distribution for consumers’ valuation, one can consider a general distribution and examine the influence of distribution variation on equilibrium outcomes. Finally, one can also extend the analysis to the cases with random demand or/and imperfect information. However, it might be outrageously complex and challenging.

## Supporting information

S1 Dataset Numerical examples. (RAR)
